# Retraction Notice, Vol. 13, August 4 Release

**DOI:** 10.5888/pcd13.160044r

**Published:** 2016-12-23

**To the Editor:**

Due to an unintentional error, the MMAS-8 scale in our article, “Health and Nutrition Literacy and Adherence to Treatment in Children, Adolescents, and Young Adults with Chronic Kidney Disease and Hypertension, North Carolina, 2015” (1), published on August 4, 2016, by *Preventing Chronic Disease,* was used without proper permission from Dr Donald E. Morisky and coauthors. We regret any problems our article may have caused, and we retract it from the literature.

**Nikita Patel, BSPH; Maria Ferris, MD; Eniko Rak, PhD**
The University of North Carolina at Chapel Hill School of Medicine
Chapel Hill, North Carolina

**Response: *PCD* marked this article as retracted on December 23, 2016.**
